# ZNPs reduce epidermal mechanical strain resistance by promoting desmosomal cadherin endocytosis via mTORC1-TFEB-BLOC1S3 axis

**DOI:** 10.1186/s12951-024-02519-z

**Published:** 2024-06-05

**Authors:** Xuan Lai, Menglei Wang, Zhen Zhang, Suya Chen, Xiner Tan, Wenjing Liu, Huimin Liang, Li Li, Longquan Shao

**Affiliations:** 1 Stomatological Hospital, School of Stomatology, Southern Medical University, Guangzhou, 510515 China; 2 Nanfang Hospital, Southern Medical University, Guangzhou, 510515 China; 3 Hospital of Stomatology, Guanghua school of Stomatology, Sun Yat-sen University, Guangzhou, 510080 China

**Keywords:** Zinc oxide nanoparticles, Epidermis, Desmosome, Fragmentation

## Abstract

Zinc oxide nanoparticles (ZNPs) are widely used in sunscreens and nanomedicines, and it was recently confirmed that ZNPs can penetrate stratum corneum into deep epidermis. Therefore, it is necessary to determine the impact of ZNPs on epidermis. In this study, ZNPs were applied to mouse skin at a relatively low concentration for one week. As a result, desmosomes in epidermal tissues were depolymerized, epidermal mechanical strain resistance was reduced, and the levels of desmosomal cadherins were decreased in cell membrane lysates and increased in cytoplasmic lysates. This finding suggested that ZNPs promote desmosomal cadherin endocytosis, which causes desmosome depolymerization. In further studies, ZNPs were proved to decrease mammalian target of rapamycin complex 1 (mTORC1) activity, activate transcription factor EB (TFEB), upregulate biogenesis of lysosome-related organelle complex 1 subunit 3 (BLOC1S3) and consequently promote desmosomal cadherin endocytosis. In addition, the key role of mTORC1 in ZNP-induced decrease in mechanical strain resistance was determined both in vitro and in vivo. It can be concluded that ZNPs reduce epidermal mechanical strain resistance by promoting desmosomal cadherin endocytosis via the mTORC1-TFEB-BLOC1S3 axis. This study helps elucidate the biological effects of ZNPs and suggests that ZNPs increase the risk of epidermal fragmentation.

**Graphical abstract:**

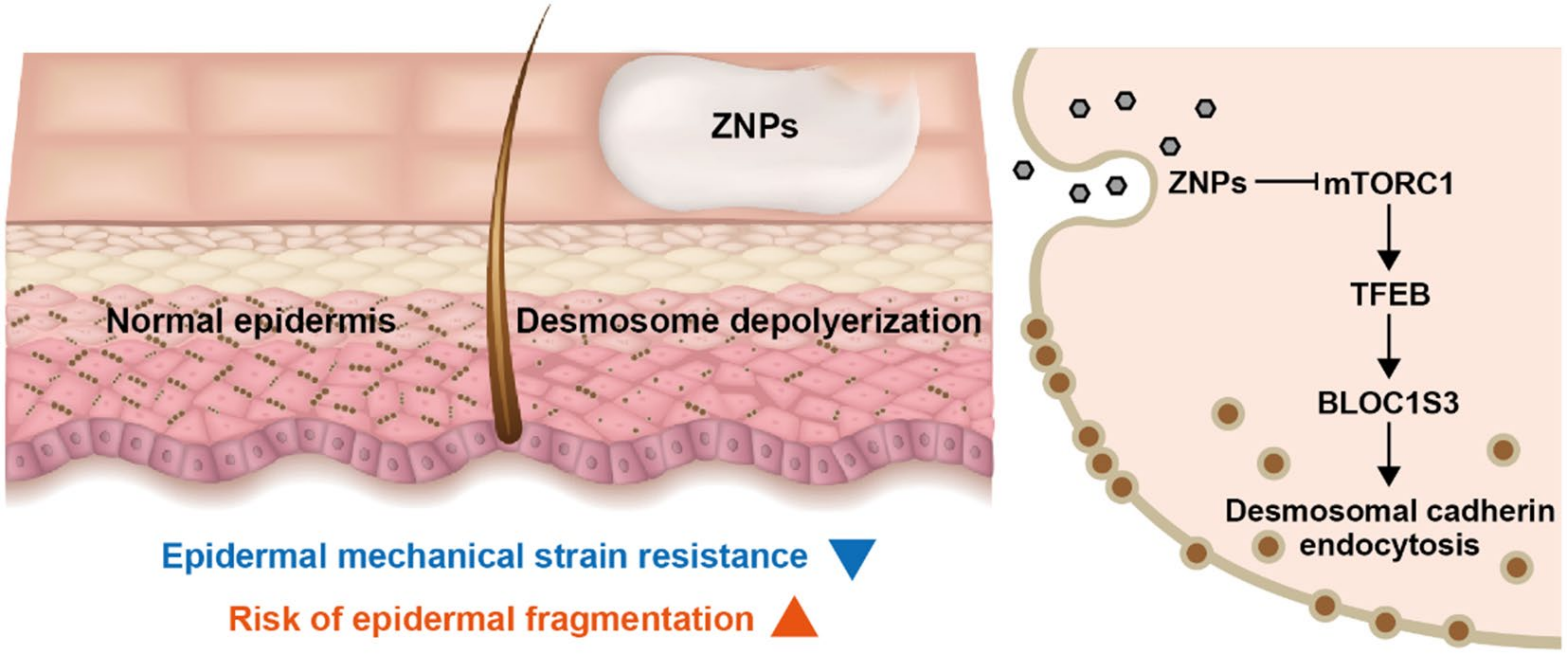

## Introduction

Zinc oxide nanoparticles (ZNPs) are multifunctional nanomaterials that can contact skin in various manners. In the field of skin care, ZNPs are excellent physical sunscreens that can also be added to cosmetics to modify skin tone. In the field of medicine, ZNPs can inhibit bacteria and promote the transdermal penetration of drugs [1–5]. The application of ZNPs to skin has long been considered safe because previous studies have shown that stratum corneum, the outermost layer of epidermal barrier, prevents ZNPs from penetrating into deep epidermis [6–9]. However, stratum corneum is susceptible to excessive cleaning or frequent rubbing, causing it to fall off. Even if stratum corneum is present, the permeability of epidermal barrier can be increased in common conditions such as sunburn, allergy, infection and inflammation. These conditions allow ZNPs to penetrate into deep epidermis. As found in an inflammatory skin model, ZNPs can penetrate stratum corneum into deep epidermis and dermis, even when the stratum corneum and epidermis are thickened [10]. However, our understanding of how ZNPs deposited in the deep epidermis affect epidermal barrier is incomplete.

The general function of epidermal barrier includes controlling the outflow of internal substances and the entry of external physicochemical irritants and microorganisms as well as providing resistance to mechanical strain [11, 12]. Mechanical strain resistance is essential for the structural and functional integrity of epidermal barrier. If mechanical strain resistance is lost, epidermis can break more easily, and the other functions of epidermal barrier are impaired. At the microscopic level, epidermis provides mechanical strain resistance through special intercellular junctions between keratinocytes called desmosomes. Desmosomes are composed of structure proteins, mainly desmosomal cadherins, armadillo proteins and plakins [13–15]. It has been reported that the levels of several cadherins between epithelial cells are reduced after ZNP application, but it is still unknown whether the levels of desmosomal cadherins are altered [16–18]. Theoretically, alterations in the levels of desmosomal cadherins or other desmosomal structural proteins are likely to reduce epidermal mechanical strain resistance and greatly increase the risk of epidermis fragmentation. However, this still needs to be demonstrated in labs.

To confirm the effect of ZNPs on desmosome and epidermal mechanical strain resistance, we applied ZNPs to mouse skin. It was found that ZNP application leads to desmosome depolymerization and desmosomal cadherin endocytosis and reduces epidermal mechanical strain resistance. While desmosomal cadherin endocytosis has been reckoned to be potentially associated with autophagy, this regulatory relationship has not been demonstrated in detail. Therefore, the molecular mechanism by which autophagy affects desmosomal cadherin endocytosis was explored in vitro. In brief, it was proven that ZNPs decreased mammalian target of rapamycin complex 1 (mTORC1) activity, thereby activating transcription factor EB (TFEB), upregulating biogenesis of lysosome-related organelle complex 1 subunit 3 (BLOC1S3) and consequently promoting desmosomal cadherin endocytosis, thus reducing the mechanical strain resistance of keratinocyte layers. This study suggests that ZNPs increase the risk of epidermal fragmentation and may provide potential targets for treating ZNP-induced epidermal damage.

## Materials and methods

### ZNP suspensions

The ZNPs (Sigma­Aldrich, USA) used in this study were the same as those used in our previous studies. The primary size of the ZNPs was 41.34 ± 9.41 nm, they were hexagonal, and their hydrodynamic size in distilled water was 266.7 ± 69.2 nm [10]. For the animal experiments, a ZNP suspension consisting of 1 mg/mL ZNPs, 0.5% HPMC and normal saline and a negative control suspension consisting of 0.5% hydroxypropyl methyl cellulose (HPMC; Aladdin, China) and normal saline were prepared. HPMC is an agent that promotes uniform dispersion of ZNPs in liquid. For the cell experiments, a ZNP suspension consisting of 1 mg/mL ZNPs and complete medium was prepared and diluted with complete medium to the desired concentration when used. The suspensions were ultrasonically dispersed in a refrigerator at 4 °C for 30 min before each use.

### Animals

Six-week-old female BALB/c mice were purchased from the Guangdong Medical Laboratory Animal Center (Guangdong, China) and were housed in an SPF animal room at the Laboratory Animal Research Center, Nanfang Hospital, Southern Medical University, after ethical approval was obtained (NFYY-2017-47). A 6-cm^2^ area of the dorsal skin was shaved. To clean the skin and remove the stratum corneum, 0.2 g of silicon dioxide scrub cream was smeared on the dorsal skin, gently massaged for 2 min and washed off using clean normal saline and gauze. Then, 100 µL of the negative control suspension or ZNP suspension was applied once daily for one week.

### Cells

The normal human immortalized keratinocytes (HaCaT cells) were obtained from the Cell Resource Center of Peking Union Medical College (Beijing, China) and were cultured in minimum Eagle’s medium (MEM) supplemented with 10% foetal bovine serum at 37 °C in a 5% CO_2_ humidified incubator. HaCaT cells were treated with complete medium or the complete medium containing 10, 20, 30 or 40 µg/mL ZNPs. The cells were harvested at 1 or 2 h intervals as needed for subsequent assays.

### Inhibitors and activators

3-Benzyl-5-(2-nitrophenoxymethyl)-γ-butyrolactone derivative (3BDO; TargetMol, USA) is an inhibitor of autophagy and an activator of mTORC1 [19–21]. Rapamycin (RAPA; TargetMol, USA) is an activator of autophagy and an inhibitor of mTORC1 [22–24]. These reagents were diluted in DMSO. In vivo, phosphate buffer solution-diluted 3BDO was applied to the skin surface at 6 h before ZNP application once daily. In vitro, cells were pretreated with 3BDO and RAPA for 3 h before ZNP treatment. An equal volume of DMSO was added as the negative control.

### Transfection

When the cells reached 60% confluence, they were transfected with a small-interfering RNA (siRNA) with ExFect Transfection Reagent (Vazyme, China) according to the manufacturer’s protocol. The siRNAs targeting TFEB and BLOC1S3 were provided by Beijing Tsingke Biotech Co., Ltd. A nontargeting siRNA (Tsingke, China) served as the negative control. After transfection, the cells were lysed and analysed by western blotting to assess the knockdown efficiency of the siRNAs.

### Transmission electron microscopy (TEM) observation

Freshly excised skin tissues (2 × 2 mm^2^) were immersed in 2.5% glutaraldehyde overnight at 4 °C. The cells were washed with 37 °C PBS and fixed with 2.5% glutaraldehyde. The fixed cells were collected and centrifuged at 1000 × g for 5 min at 4 °C. Then, the samples were embedded in resin, cut into ultrathin slices and stained with osmic acid. The microstructure of desmosomes between keratinocytes was observed using TEM (Hitachi, Japan).

### Dissociation assay

Freshly excised skin tissues were immersed in 0.25% dispase II (Sigma­Aldrich, USA) overnight at 4 °C. Cells were washed with Hank’s balanced salt solution (HBSS) and immersed in 0.25% dispase II for 5 min at 37 °C. Dispase solution was then replaced with HBSS. The epidermal tissues were carefully separated. The keratinocytes were carefully scraped together. Mechanical stress was applied by an electric pipette (Eppendorf, Germany) of 1 mL pipetting up and down for 5 times. The fragments of epidermis tissues and keratinocyte monolayers were counted.

### Immunohistochemistry (IHC) and fluorescence observation

Freshly excised skin tissues were immersed in 4% paraformaldehyde for 24 h and embedded in paraffin wax. Tissue slices were prepared, and an IHC assay and fluorescence observation was performed. The expression levels of desmosomal structural proteins in the epidermis were measured with antibodies against the desmogleins Dsg1 (Proteintech, USA) and Dsg3 (Santa Cruz, USA) and the desmocollins Dsc1 (Santa Cruz, USA) and Dsc3 (Santa Cruz, USA), desmoplakin (DP; CST, USA), plakoglobin (PG; Santa Cruz, USA) and plakophilin (PKP2; Proteintech, USA). HaCaT cells were seeded on slides, treated with ZNPs, stained with a cell membrane probe (Beyotime, China), washed with PBS after staining and fixed in 4% paraformaldehyde overnight. The fixed cells were permeabilized with 0.5% Triton X-100 for 30 min, blocked with 5% FBS for 1 h, incubated with primary antibodies against pan-keratin (Proteintech, USA), Dsg1 (Proteintech, USA), Dsg3 (Santa Cruz, USA), Dsc1 (Santa Cruz, USA), Dsc3 (Santa Cruz, USA), LC3B (Bimake, USA; Santa Cruz, USA), TFEB (Santa Cruz, USA) and BLOC1S3 (Santa Cruz, USA) at 4 °C overnight and incubated with fluorochrome-conjugated secondary antibodies (Proteintech, USA) at room temperature for 1 h. Images were captured using a microscope (Leica, Germany).

### Western blot

Cells or epidermal tissues were harvested and lysed using commercial kits (Invent, USA). The lysates of whole cells, cell membranes, cytoplasm and cell nucleus were obtained according to the manufacturer’s protocols, mixed with 5× sodium dodecyl sulphate‒polyacrylamide gel electrophoresis sample buffer (Genstar, China) and heated at 99 °C for 5 min. The proteins were separated by sodium dodecyl sulphate‒polyacrylamide gel electrophoresis and transferred to polyvinylidene difluoride membranes (Millipore, Germany). The membranes were blocked with a rapid blocking solution (Beyotime, China) at room temperature for 15 min, incubated overnight at 4 °C with primary antibodies against Dsg1 (Proteintech, USA), Dsg3 (Santa Cruz, USA), Dsc1 (Santa Cruz, USA), Dsc3 (Santa Cruz, USA), DP (CST, USA), PG (Santa Cruz, USA), PKP2 (Proteintech, USA), ribosomal protein S6 kinase (S6K; ABclonal, China), phospho-S6K (p-S6K; ABclonal, China), sequestosome 1 (SQSTM1; Santa Cruz, USA), microtubule-associated protein 1 light chain 3 beta (LC3B; Bimake, USA; Santa Cruz, USA), TFEB (Santa Cruz, USA), phospho-TFEB (p-TFEB; CST, USA) and BLOC1S3 (Santa Cruz, USA) and incubated at room temperature with horseradish peroxidase-conjugated secondary antibodies (CST, USA) for 1 h. The target protein bands were visualized using an ECL kit (EpiZyme, China) and an automatic chemiluminescence image analysis system (Tanon, China).

### Proteomic analysis

Epidermal tissues were collected and lysed in SDT buffer (Leigen, China) at low temperature. The lysates were centrifuged at 12,000 × g and 4 °C. The supernatants were obtained and mixed with 5 mM DTT at 56 °C for 30 min. Then, 100 mM iodoacetamide was added, and the mixture was mixed for 30 min in the dark. The mixtures were treated with trypsin at 37 °C for 16 h. The polypeptides were labelled using a commercial TMT kit (Thermo Fisher Scientific, USA). LC‒MS/MS analysis was performed using a timsTOF Pro mass spectrometer (Bruker, Germany). The raw MS data were combined and searched using MaxQuant software. The sequences of the differentially expressed proteins and the sequences of the differentially phosphorylated proteins were determined using NCBI BLAST + client and InterProScan to find homologous sequences. The proteins were searched against the online Kyoto Encyclopedia of Genes and Genomes (KEGG) database and annotated with associated pathways. Enrichment analysis was performed based on Fisher’s exact test. All quantified proteins were considered the background dataset. Benjamini‒Hochberg correction for multiple tests was applied to adjust the derived p values. A functional category or pathway was considered significantly enriched only when *P* < 0.05.

### Statistical analysis

All the data were analysed with GraphPad Prism 9 software. Comparisons among the groups were assessed using one-way ANOVA when the variance was homogenous or a nonparametric test when the variance was not homogenous. Differences for which *P* < 0.05 were considered to be statistically significant.

## Results

### ZNPs cause desmosome depolymerization and reduce epidermal mechanical strain resistance

**Fig. 1:**
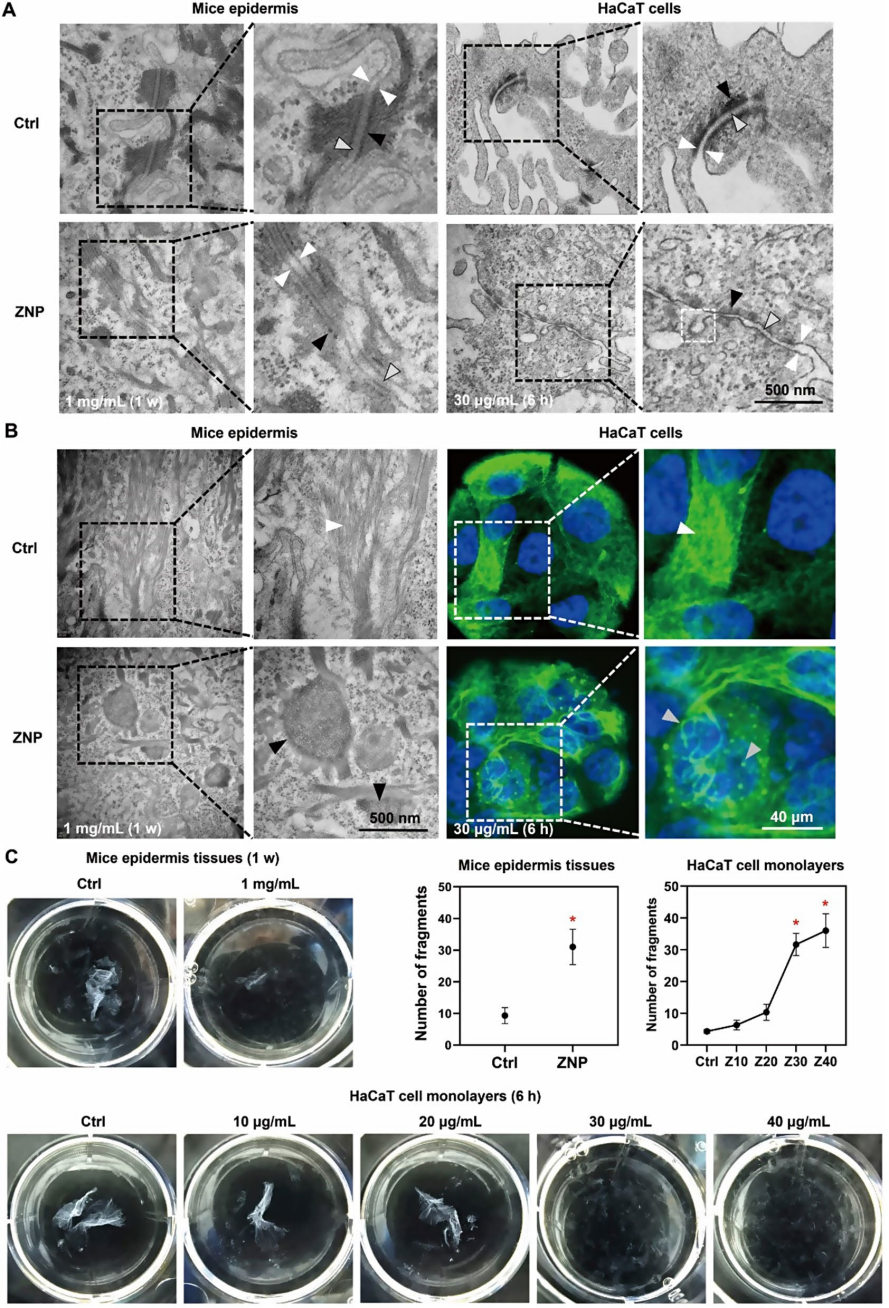
ZNPs cause desmosome depolymerization and reduce epidermal mechanical strain resistance. (**A**) TEM images showing that ZNP application (1 mg/mL for 1 week) resulted in depolymerization of desmosomes in mouse epidermal tissues; ZNP treatment (30 µg/mL for 6 h) resulted in depolymerization of desmosomes between HaCaT cells. The white arrows indicate cell membrane, the black arrows indicate desmosomal cytoplasmic plaque, the gray arrows indicate desmosomal extracellular plaque, and the dotted white square indicate endocytic vesicle. (**B**) TEM images of mouse epidermal tissues showed that the intermediate fibres were uniformly arranged in the same direction as those in the control group, and collapsed and aggregated into beads after ZNP application (1 mg/mL for 1 week). Fluorescence images of HaCaT cells showed similar results after ZNP treatment (30 µg/mL for 6 h). The white arrows show normal and uniform intermediate fibres; the black arrows and the gray arrows show collapsed and abnormally aggregated intermediate fibres. (**C**) According to the dissociation assay, the number of fragments of mouse epidermal tissues was increased after ZNP treatment (1 mg/mL for 1 week), and the number of fragments of HaCaT cell monolayers was increased after ZNP treatment (30 and 40 µg/mL for 6 h) (*P < 0.05, *n* = 3)

ZNP application to the skin of mice resulted in alterations in the microstructure of desmosomes between epidermal keratinocytes. TEM images of desmosomes showed that in the control group, the extracellular plaques were uniform and dense, and the cytoplasmic plaques were close to cell membrane. In ZNP group, the electron density of desmosomes was decreased, parts of the extracellular plaques and most of the cytoplasmic plaques were lost, and the residual cytoplasmic plaques were detached from cell membrane. Similar results were observed in vitro (Fig. 1A). This suggested that ZNPs caused desmosome depolymerization.

In addition to adjacent cell membranes, desmosomes also anchor intermediate fibres to cell membrane. Desmosome depolymerization can result in the collapse and abnormal aggregation of intermediate fibres [13–15]. TEM images of mouse epidermal tissues and fluorescence images of HaCaT cells showed that the intermediate fibres in the control group were uniformly arranged in the same direction, while the intermediate fibres in ZNP group collapsed and aggregated into beads (Fig. 1B). These results were consistent with ZNP-induced desmosome depolymerization.

According to the dissociation assays, ZNP application reduced the mechanical strain resistance of mouse epidermis and keratinocyte monolayers. In the negative control group, the epidermis and keratinocyte monolayer were relatively intact after mechanical stress was applied. In ZNP group, it was difficult to separate an intact epidermis from dermis, and the epidermis became more fragmented under mechanical stress. Similar results were observed in vitro (Fig. 1C). These results suggested that ZNPs decreased epidermal mechanical strain resistance and increased epidermal fragmentation.

### ZNPs promote desmosomal cadherin endocytosis, which leads to desmosome depolymerization

**Fig. 2:**
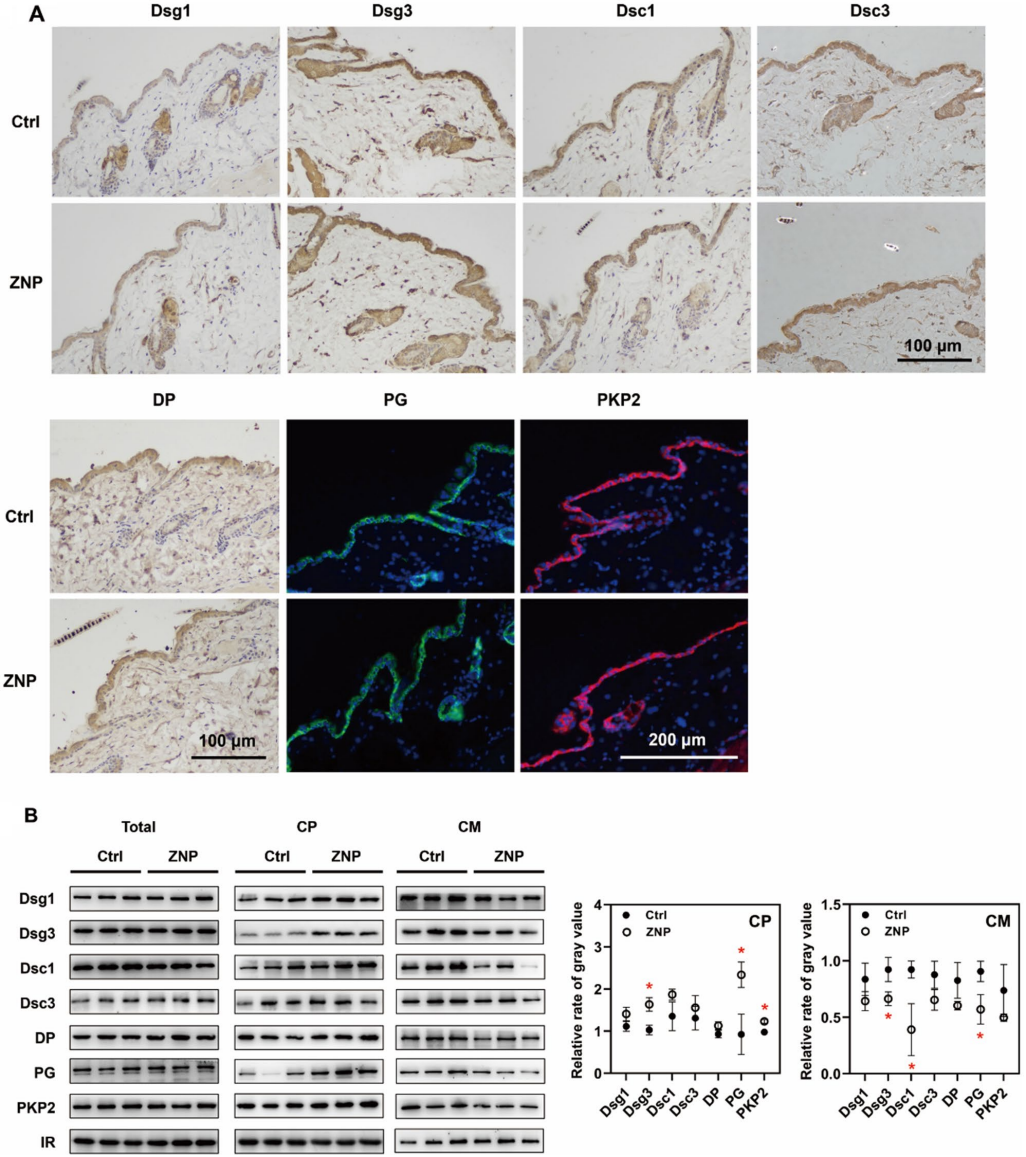
ZNPs promoted the endocytosis of desmosomal cadherins and other structural proteins in vivo. (**A**) IHC results showed that the levels of Dsg1, Dsg3, Dsc1, Dsc3, DP, PG and PKP2 were not obviously changed after ZNP treatment (1 mg/mL for 1 week). (**B**) Immunoblotting showed that the levels of Dsg1, Dsg3, Dsc1, Dsc3, DP, PG and PKP2 in whole cell lysates were not significantly changed, the levels of Dsg3, PG and PKP2 in cytoplasmic lysates were significantly increased, and the levels of Dsg3, Dsc1 and PG in cell membrane lysates were significantly decreased after ZNP application. (ZNP: 1 mg/mL for 1 week; Total: whole cell lysates; CP: cytoplasmic lysates; CM: cell membrane lysates; IR in Toal and CP: GAPDH; IR in CM: Na/K ATPase; *P < 0.05, *n* = 3.)

**Fig. 3:**
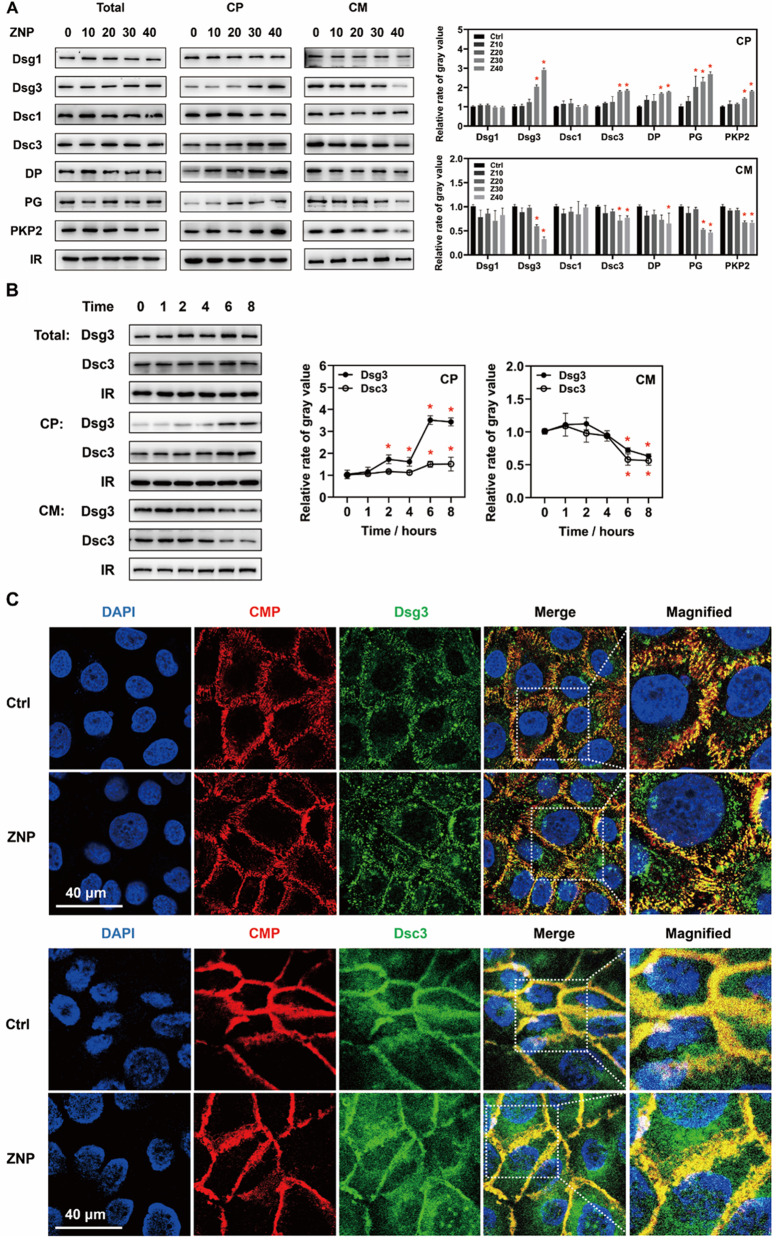
ZNPs promoted the endocytosis of desmosomal cadherins and other structural proteins in vitro. (**A**) Immunoblotting showed that the levels of Dsg1, Dsg3, Dsc1, Dsc3, DP, PG and PKP2 in whole cell lysates were not significantly changed; the levels of Dsg3, Dsc3, DP, PG and PKP2 in cytoplasmic lysates were significantly increased; the levels of Dsg3, Dsc3, DP, PG and PKP2 in cell membrane lysates were significantly decreased after ZNP treatment (30 or 40 µg/mL for 6 h). (**B**) In cytoplasmic lysates, a significant increase in the levels of Dsg3, Dsc3, DP, PG and PKP2 were observed beginning 2 h after ZNP treatment. In cell membrane lysates, a significant decrease in the levels of these proteins was observed beginning 6 h after ZNP treatment. (**C**) Fluorescence images showed that after ZNP treatment (30 µg/mL for 6 h), more green fluorescence of Dsg3 and Dsc3 were not colocalized with the red fluorescence of cell membrane, and instead were in cytoplasm. (Total: whole cell lysates; CP: cytoplasmic lysates; CM: cell membrane lysates; IR in Toal and CP: GAPDH; IR in CM: Na/K ATPase; CMP: cell membrane probes; *P < 0.05, *n* = 3)

The expression levels of desmosomal structural proteins were quantified. The levels of Dsg1, Dsg3, Dsc1, Dsc3, DP, PG and PKP2 in whole cell lysates were not decreased in vivo or in vitro after ZNP application. The levels of these proteins in cytoplasmic lysates and cell membrane lysates were subsequently measured. Immunoblotting in vivo showed that the levels of Dsg3, PG and PKP2 in cytoplasmic lysates were increased, while the levels of Dsg3, Dsc1 and PG in cell membrane lysates were decreased (Fig. 2). In vitro, the levels of Dsg3, Dsc3, DP, PG and PKP2 were increased in cytoplasmic lysates and decreased in cell membrane lysates when the concentration of ZNPs was 30 or 40 µg/mL (Fig. 3A). In cytoplasmic lysates, a significant increase in the levels of Dsg3, Dsc3, DP, PG and PKP2 were observed beginning 2 h after ZNP treatment. A significant decrease in the levels of these proteins was observed in cell membrane lysates beginning at 6 h after ZNP treatment (Fig. 3B). The above results suggested that ZNPs promote the translocation of desmosomal structural proteins from cell membrane to cytoplasm.

According to fluorescence analysis, the green fluorescence of Dsg3 and Dsc3 and the red fluorescence of cell membrane were mostly colocalized in untreated HaCaT cells. However, in ZNP-treated HaCaT cells, many green fluorescent signals of Dsg3 and Dsc3 were not colocalized with the red fluorescence signals of cell membrane, and instead were in cytoplasm (Fig. 3C). These findings suggested that ZNPs promote the localization of Dsg3 and Dsc3 in cytoplasm.

**Fig. 4:**
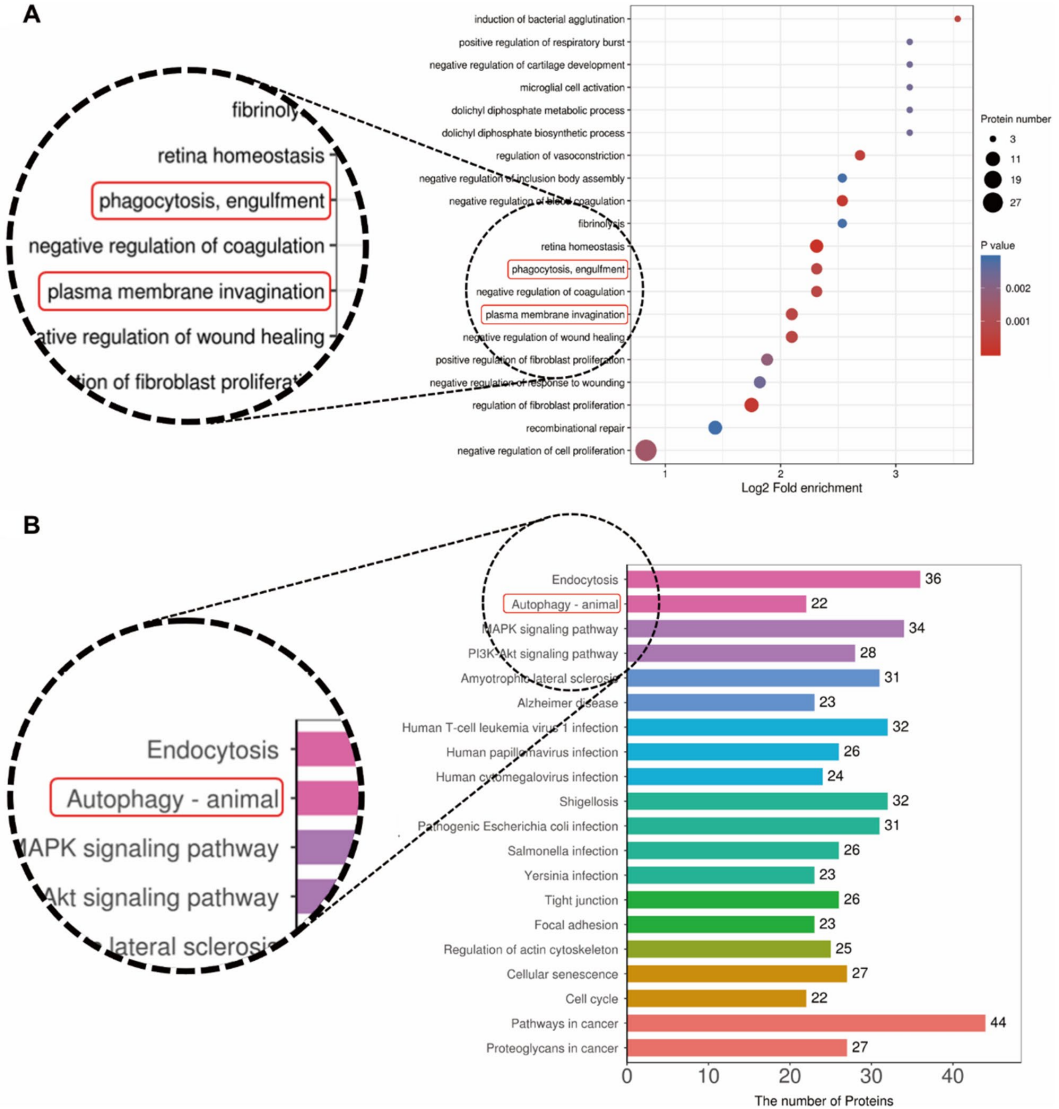
Proteomic analysis of the KEGG database. (**A**) The ZNP-upregulated proteins were enriched in processes related to endocytosis. (**B**) The ZNP-induced differentially expressed proteins were enriched in processes related to the autophagy. (*P* < 0.05; *n* = 6)

According to proteomic analysis, the ZNP-upregulated proteins were enriched in processes related to phagocytosis, engulfment and plasma membrane invagination (Fig. 4A). This finding suggested an increase in cellular endocytosis. TEM images also showed that endocytic vesicles appeared near the depolymerized desmosomes (Fig. 1A). Therefore, it can be speculated that ZNPs promote desmosomal cadherin endocytosis, which causes desmosome depolymerization.

### ZNPs cause desmosomal cadherin endocytosis and decreased mechanical strain resistance by decreasing mTORC1 activity

**Fig. 5:**
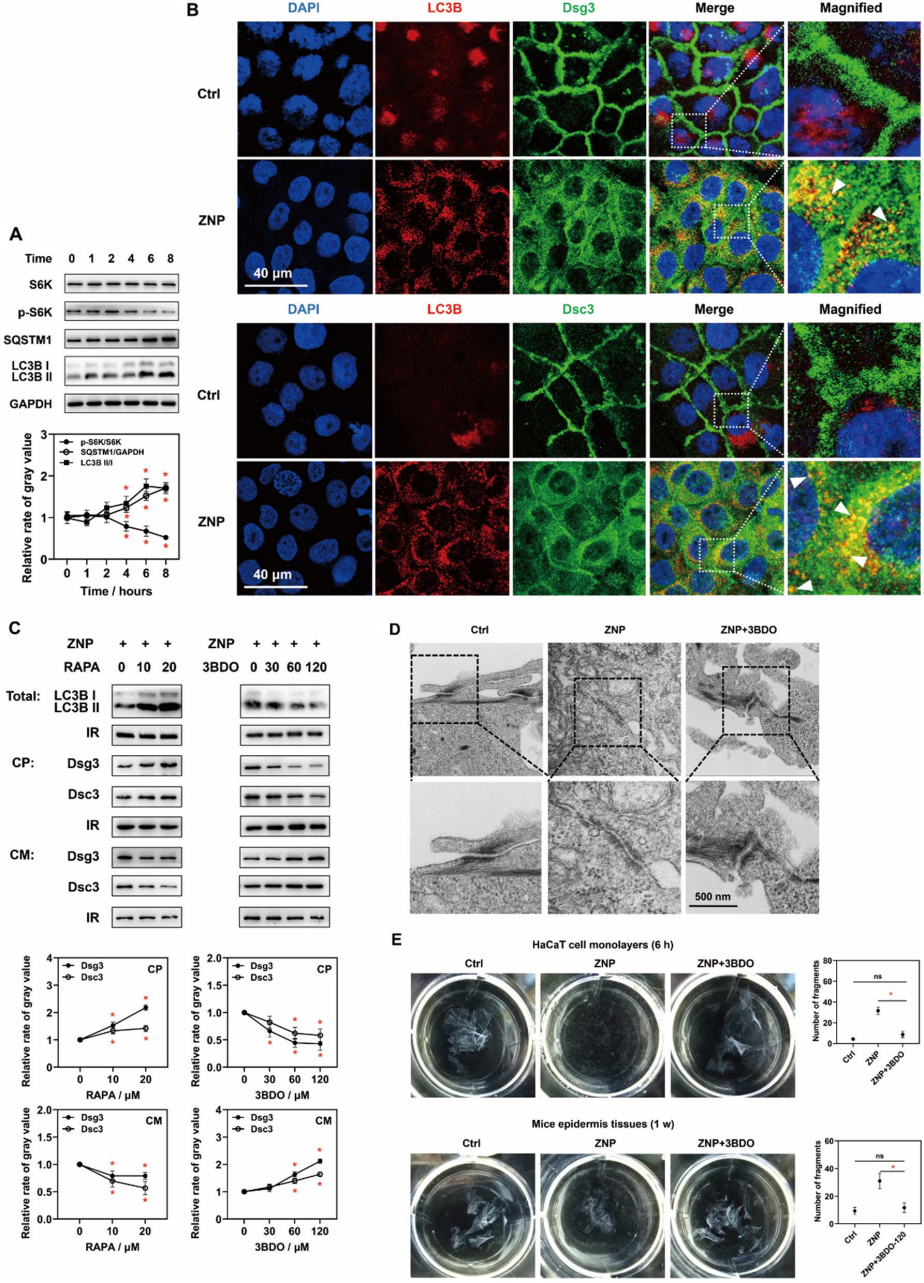
Roles of autophagy and mTORC1 in ZNP-induced desmosomal cadherin endocytosis. (**A**) Immunoblotting showed that S6K phosphorylation and SQSTM1 level were significantly decreased and that LC3B II/I ratio was increased after ZNP treatment (30 µg/mL for 4 h at least) (*P < 0.05, *n* = 3). (**B**) Fluorescence images showed that ZNP treatment (30 µg/mL for 6 h) promoted Dsg3 and Dsc3 partially colocalized with LC3B in cytoplasm. (**C**) Inhibiting autophagy by using an activator of mTORC1, 3BDO (60 and 120 µM), rather than promoting autophagy by using an inhibitor of mTORC1, RAPA (10 and 20 µM), was effective at reversing ZNP-induced translocation of Dsg3 and Dsc3 from cell membrane to cytoplasm (Total: whole cell lysates; CP: cytoplasmic lysates; CM: cell membrane lysates; IR in Toal and CP: GAPDH; IR in CM: Na/K ATPase; *P < 0.05, *n* = 3). (**D**) TEM images showed that 3BDO (120 µM) prevented ZNP-induced desmosome depolymerization. (**E**) According to dissociation assays, 3BDO (120 µM) was effective at reversing ZNP-induced fragmentation of HaCaT cell monolayers and mouse epidermal tissues (in vitro: 30 µg/mL ZNPs for 6 h; in vivo: 1 mg/mL ZNPs for 1 week; *P < 0.05, *n* = 3)

Autophagy was speculated to be associated with the endocytosis of desmosomal structural proteins in a previous study [15]. According to proteomic analysis, the ZNP-induced differentially expressed proteins were enriched in processes related to autophagy (Fig. 4B). Inmmunoblots revealed that at 6 h after 30 µg/mL ZNP treatment, the phosphorylation of S6K was significantly decreased. S6K is a substrate of mTORC1. The decreased phosphorylation of S6K suggested reduced mTORC1 activity and autophagy initiation [19–21]. Additionally, SQSTM1 level and LC3B II/I ratio were increased (Fig. 5A). These results suggested that ZNPs promote autophagosome formation and disrupt autophagic flux [22–24].

According to fluorescence analysis, the green fluorescence of Dsg3 and Dsc3 and the red fluorescence of LC3B were not colocalized in untreated cells. However, in ZNP-treated cells, the green fluorescent signals of Dsg3 and Dsc3 were partially colocalized with the red fluorescent signals of LC3B. In addition, the intensity of red fluorescence of LC3B was greater in ZNP-treated cells than in untreated cells (Fig. 5B). These findings suggested that ZNPs promote autophagy, which may be associated with desmosomal cadherin endocytosis.

To determine the roles of autophagy and mTORC1 in this effect, 3BDO, an activator of mTORC1, was used to inhibit autophagy, and RAPA, an inhibitor of mTORC1, was used to activate autophagy [25–30]. The immunoblot results showed that 3BDO but not RAPA successfully reversed the increase in LC3BII/I ratio, the increase in the levels of Dsg3 and Dsc3 in cytoplasmic lysates and the decrease in the levels of Dsg3 and Dsc3 in cell membrane lysates in the presence of ZNPs (Fig. 5C). This result suggested that ZNP-induced desmosomal cadherin endocytosis was attributed to reduced mTORC1 activity. Consistently, 3BDO efficiently prevented ZNP-induced desmosome depolymerization and fragmentation of keratinocyte monolayers (Fig. 5D and E). Addtionally, in vivo, 3BDO reduced ZNP-induced epidermal fragmentation (Fig. 5E).

### ZNP-induced decrease in mTORC1 activity leads to TFEB activation, which promotes desmosomal cadherin endocytosis and decreased mechanical strain resistance

**Fig. 6:**
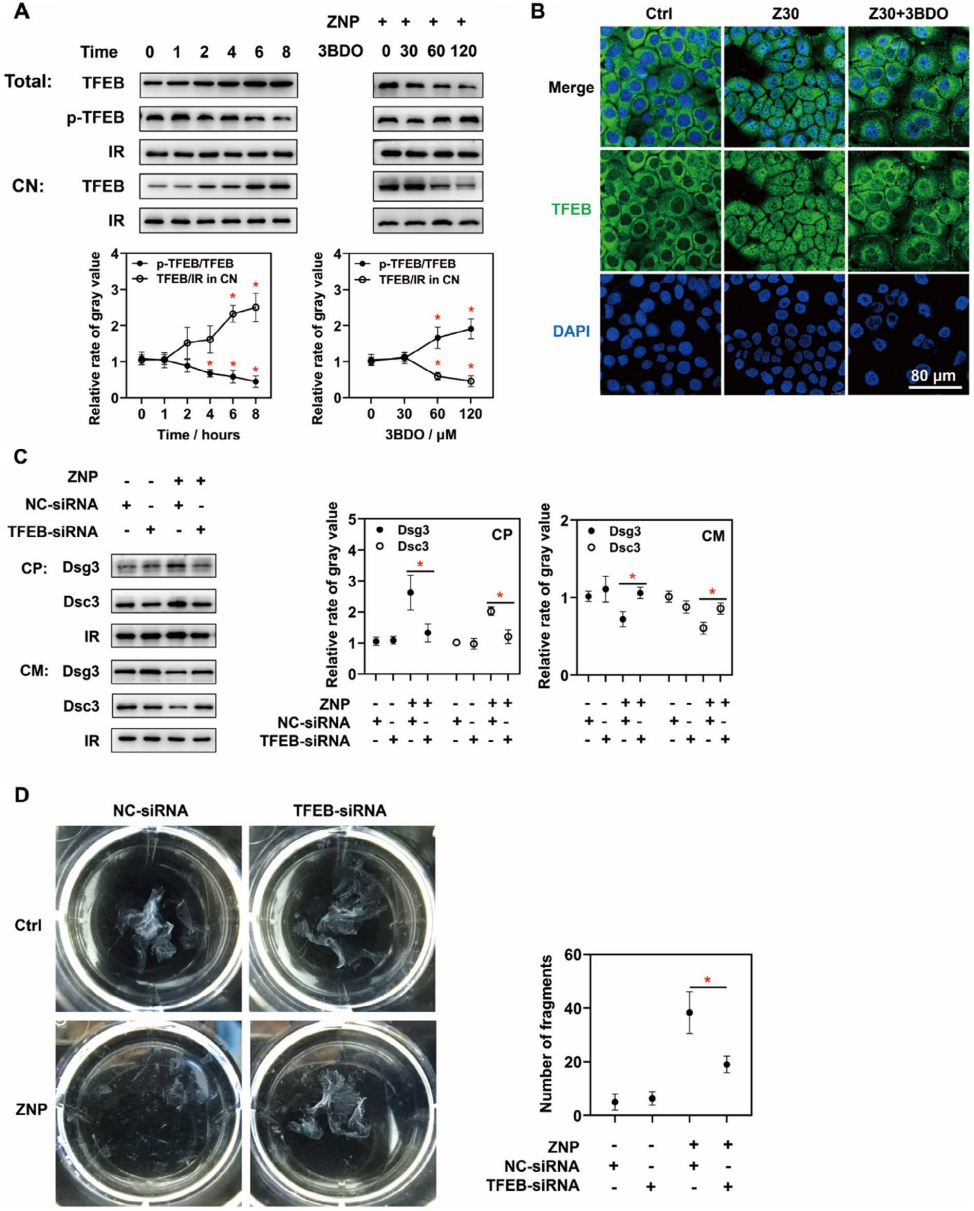
Roles of TFEB in ZNP-induced desmosomal cadherin endocytosis. (**A**) Immunoblotting showed that TFEB phosphorylation was significantly decreased and that TFEB level in nuclear lysates was significantly increased after ZNP treatment (30 µg/mL for 4 h at least). These changes were inhibited by 3BDO at doses of 60 and 120 µM. (**B**) Fluorescence images also showed that ZNP treatment (30 µg/mL for 6 h) promoted TFEB translocation into nucleus, which was inhibited by 3BDO at the dose of 120 µM. (**C**) Immunoblotting results showed that the levels of Dsg3 and Dsc3 were decreased in cytoplasmic lysates and increased in cell membrane lysates in TFEB-siRNA group than in NC-siRNA group in the presence of ZNPs (30 µg/mL for 6 h). (**D**) According to dissociation assays, there were fewer fragments in TFEB-siRNA group than in NC-siRNA group. (Total: whole cell lysates; CN: cell nuclear lysates; CP: cytoplasmic lysates; CM: cell membrane lysates; IR in Toal and CP: GAPDH; IR in CN: TBP; IR in CM: Na/K ATPase; *P < 0.05, *n* = 3)

According to the proteomic analysis of the phosphorylated proteins, the level of phosphorylated TFEB was drastically decreased by ZNPs. TFEB is known as a downstream transcription factor of mTORC1 and can be activated by decreased mTORC1 activity. Recently reported, TFEB activation promotes the expression of genes related to endocytosis [31–35]. Immunoblotting showed that ZNP treatment decreased the p-TFEB/TFEB ratio in whole cell lysates and increased TFEB levels in nuclear lysates. Fluorescence images showed that abundant green fluorescence of TFEB, was present in nucleus after ZNP treatment (Fig. 6A). These results proved that ZNPs activated TFEB. When 3BDO was used to increase mTORC1 activity, the p-TFEB/TFEB ratio in whole cell lysates was increased, the TFEB level in nuclear lysates decreased, and the green fluorescent signals of TFEB in nucleus almost disappeared (Fig. 6B). This result suggested that ZNP-induced decrease in mTORC1 activity leads to TFEB activation.

Furthermore, TFEB-siRNA was used to inhibit TFEB expression. Compared with the nontargeting siRNA, the TFEB-siRNA obviously decreased the levels of Dsg3 and Dsc3 in cytoplasmic lysates and increased the levels of Dsg3 and Dsc3 in cell membrane lysates in the presence of the ZNPs (Fig. 6C). Consistent with these findings, fewer fragments of keratinocyte monolayers were observed in TFEB-siRNA group (Fig. 6D). The above results suggested that ZNP-induced TFEB activation promoted desmosomal cadherin endocytosis and reduced the mechanical strain resistance of keratinocyte monolayers.

### TFEB-induced BLOC1S3 upregulation participates in increasing desmosomal cadherin endocytosis and decreasing mechanical strain resistance

**Fig. 7:**
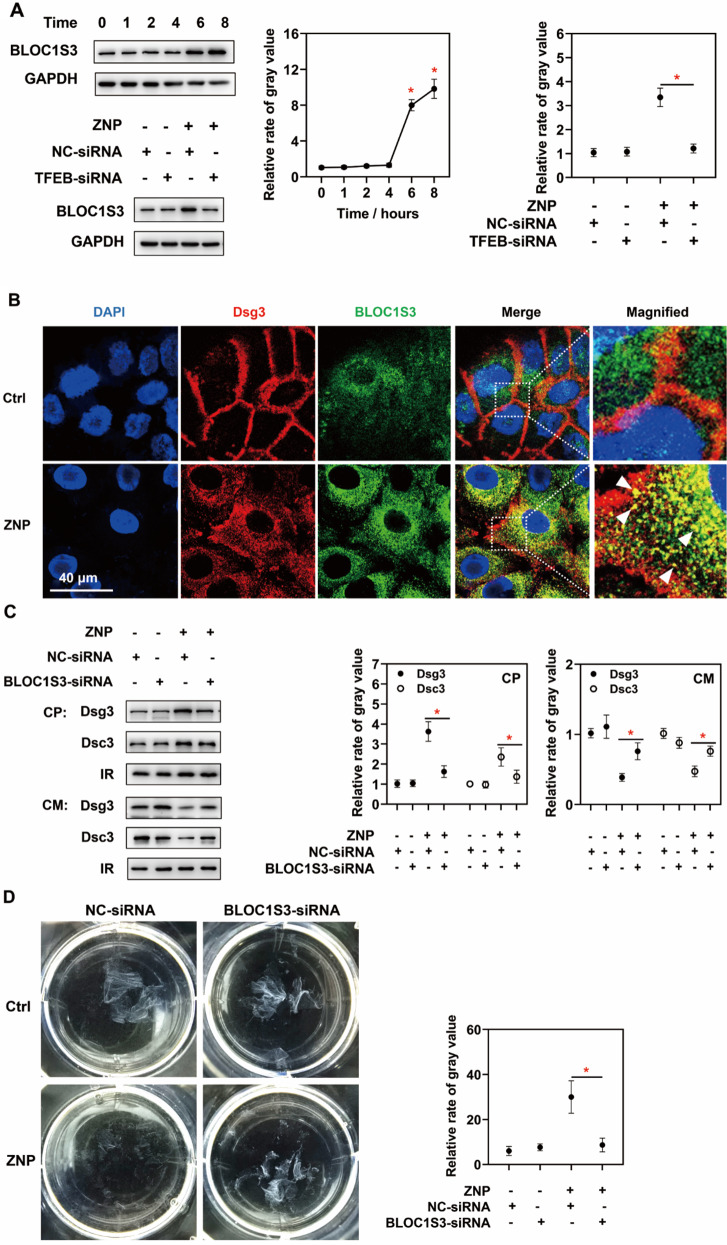
Roles of BLOC1S3 in ZNP-induced desmosomal cadherin endocytosis. (**A**) Immunoblotting showed that the BLOC1S3 level was significantly increased after ZNP treatment (30 µg/mL for 6 h at least), and which was inhibited by TFEB-siRNA. (**B**) Fluorescence images showed that ZNP treatment (30 µg/mL for 6 h) promoted BLOC1S3 colocalized with Dsg3. (**C**) Immunoblotting showed that the levels of Dsg3 and Dsc3 were decreased in cytoplasmic lysates and increased in cell membrane lysates in BLOC1S3-siRNA group than in NC-siRNA group in the presence of ZNPs (30 µg/mL for 6 h). (**D**) According to dissociation assays, there were fewer fragments of BLOC1S3-siRNA-treated cell monolayers than of NC-siRNA-treated cell monolayers. (CP: cytoplasmic lysates; CM: cell membrane lysates; IR in CP: GAPDH; IR in CM: Na/K ATPase; *P < 0.05, *n* = 3)

The potential target genes of TFEB related to endocytosis were predicted and summarized previously [33]. According to proteomic analysis, BLOC1S3 was the only gene that matched the previous prediction and was upregulated by ZNPs, and its expression increased up to 100-fold. Immunoblotting also showed an increased BLOC1S3 level. TFEB-siRNA successfully prevented the increase in BLOC1S3 levels (Fig. 7A). These findings suggested that ZNP-induced TFEB activation resulted in the upregulation of BLOC1S3.

According to fluorescence analysis, the red fluorescence of Dsg3 and the green fluorescence of BLOC1S3 were not colocalized in untreated cells. However, in ZNP-treated cells, the red fluorescent signals of Dsg3 in cytoplasm were mostly colocalized with the green fluorescent signals of BLOC1S3. In addition, the intensity of the green fluorescence of BLOC1S3 was greater in ZNP-treated cells than in untreated cells (Fig. 7B). These findings also suggested that ZNPs promote BLOC1S3 expression, which may be potentially associated with desmosomal cadherin endocytosis.

Furthermore, a siRNA was used to inhibit BLOC1S3 expression. Immunoblotting also revealed that BLOC1S3-siRNA inhibited the increase in Dsg3 and Dsc3 levels in cytoplasmic lysates and the decrease in Dsg3 and Dsc3 levels in cell membrane lysates in the presence of ZNPs (Fig. 7C). This result suggested that BLOC1S3 participated in ZNP-induced desmosomal cadherin endocytosis. This further confirmed the role of BLOC1S3 in ZNP-induced desmosomal cadherin endocytosis. According to dissociation assays, BLOC1S3-siRNA partially reduced the fragmentation of keratinocyte monolayers (Fig. 7D). These findings suggested that BLOC1S3 participated in ZNP-induced decrease in mechanical strain resistance.

## Discussion

This study confirmed that ZNPs impair epidermal mechanical strain resistance. Unlike previous studies, which have focused mostly on the relationship between ZNPs and skin inflammation, this study focused on epidermal barrier function, which is important but was previously ignored [36–40]. Considering the extensive application of ZNPs and the fact that several barrier structure proteins have been reported to be differentially expressed after ZNP application, we decided to investigate how ZNPs affect epidermal barrier function. The general function of the epidermal barrier is to control the entry and exit of substances and to provide resistance to physicochemical stimuli and mechanical forces [11, 12]. In particular, the function of epidermal barrier in providing resistance to mechanical forces is fundamentally important. Without this resistance, the epidermis becomes easily broken or even detached. In a mild case, such loss of resistance may result in local skin sensitization and inflammation; however, patients with a severe case, such as Butterfly Girl, may suffer from epidermolysis [41–45]. In a previous study, ZNPs were shown to widen the gap between epidermal keratinocytes, increasing the probability of skin sensitization and inflammation [10]. In our study, TEM images showed that ZNPs caused desmosome depolymerization, and the results of dissociation assays confirmed that ZNPs decreased epidermal mechanical strain resistance. Application of ZNPs even at the low dose (1 mg/mL; equivalent to 0.0167 mg/cm^2^) for one week resulted in a statistically significant decrease in epidermal mechanical strain resistance. The lowest effective dose of ZNPs uesed as a sunscreen is 2 mg/cm^2^ [46]. Therefore, ZNPs are unsafe for use as sunscreens. Although the exposure dose may not reach the lowest effective dose of ZNPs used as sunscreens, the relatively low dose of 0.0167 mg/cm^2^ ZNPs is enough to have a negative effect. In addition, according to another study, ZNPs can react with other components in sunscreens to produce toxic chemicals during UVB exposure [47]. However, ZNPs have long been thought to be safe and widely used in sunscreens and cosmetics, so it is necessary to clarify how ZNPs deposited in the deep epidermis might affect the health and function of epidermis.

Desmosomes between keratinocytes are known to confer mechanical strain resistance to epidermis [13]. According to TEM results, the extracellular plaques of desmosomes were partially lost, and the cytoplasmic plaques detached from cell membrane, which suggested desmosome depolymerization. We observed not only desmosome depolymerization but also abnormal aggregation of intermediate fibres. Under normal conditions, intermediate fibres are anchored at cell membrane by desmosomes, but after ZNP application, they collapsed and aggregated in a disorderly manner. This phenomenon further confirmed the abnormality of desmosomes. Intermediate fibres are also the main structures that maintain cell stiffness and play a key role in the proliferation and motility of keratinocytes [48–50]. Therefore, in addition to mechanical strain resistance, ZNPs may also reduce epidermal wound healing and spontaneous epidermal renewal.

In a futher study, to definite the alterations in desmosomes, the expression and localization of desmosomal structural proteins including desmosomal cadherins, armadillo proteins and plakins were detected. Desmosomal cadherins bind to each other in pairs and anchor adjacent cells together with the assistance of armadillo proteins and plakins [14, 15]. In TEM images, the extracellular regions of desmosomal cadherins compose the extracellular plaques of desmosomes, and the intracellular regions of desmosomal cadherins and armadillo proteins as well as plakins compose the cytoplasmic plaques that stick to cell membrane. Therefore, the loss of desmosomal extracellular plaques and cytoplasmic plaques suggested the detachment of desmosomal cadherins and other desmosomal structural proteins from the cell membrane. The immunoblot results showed that the levels of the desmosomal cadherins Dsg1, Dsg3, Dsc1 and Dsc3 and the other desmosomal structural proteins DP, PG and PKP2 in whole cell lysates were not changed in either mouse epidermal tissues or HaCaT cells. Nevertheless, in vivo, the levels of Dsg3, PG and PKP2 in cytoplasmic lysates were significantly increased, and the levels of Dsg3, Dsc1 and PKP2 in cell membrane lysates were decreased. In vitro, the levels of Dsg3, Dsc3, DP, PG and PKP2 were increased in cytoplasmic lysates and decreased in cell membrane lysates. In addition, the increase in Dsg3 and Dsc3 location from cell membrane to cytoplasm was observed via fluorescence. Moreover, proteomic analysis revealed that the proteins upregulated by ZNPs were enriched in endocytosis-related processes. These findings indicate that ZNPs promote desmosomal cadherin endocytosis, which leads to desmosome depolymerization and reduces epidermal mechanical strain resistance.

Although the types of desmosomal cadherins with altered localization in vivo and in vitro are different, this does not hinder the conclusion. These differences may have occurred because cultured keratinocytes are not exactly the same as keratinocytes in vivo. Keratinocytes grow in a three-dimensional environment and are in different states of differentiation in vivo, while cultured keratinocytes grow in a two-dimensional environment and are immortalized [51–53]. Nevertheless, the above results still proved that ZNPs promote desmosomal cadherin endocytosis.

At present, few studies have explored the mechanism by which nanomaterials induce desmosomal cadherin endocytosis. However, autophagy was speculated to be associated with the endocytosis of desmosomal structural proteins [54]. Consistent with these findings, the proteomic analysis suggested that ZNPs induced the enrichment of autophagy-related pathways. Thus, is autophagy responsible for ZNP-induced endocytosis of desmosomal cadherins? In another experiment, decreased phosphorylation of S6K was detected. S6K is a downstream substrate of mTORC1 [25]. Decreased phosphorylation of S6K indicates decreased mTORC1 activity, which initiates autophagy. Consistently, LC3BII/I ratio and the colocalization of desmosomal cadherins and LC3B were increased in the presence of ZNPs. These findings indicate that ZNPs promote autophagosome formation and the localization of desmosomal cadherins to autophagosomes. However, SQSTM1 level was increased. This generally suggests disrupted autophagic flux [22]. However, whether autophagy or the disrupted autophagic flux promotes ZNP-induced desmosomal cadherin endocytosis is unclear. Therefore, a rescue experiment is needed to determine the role of autophagy in the effect of ZNPs. 3BDO and RAPA were used to inhibit and activate autophagy, respectively. 3BDO but not RAPA decreased the ZNP-induced endocytosis of Dsg3 and Dsc3; hence, it was confirmed that ZNP-induced autophagy promotes desmosomal cadherin endocytosis. Moreover, 3BDO prevented ZNP-induced desmosome depolymerization and fragmentation of the mouse epidermis and keratinocyte monolayers in dissociation assays. Therefore, ZNPs cause desmosomal cadherin endocytosis and decreased epidermal mechanical strain resistance by promoting autophagy. However, 3BDO inhibits autophagy by directly upstream of mTORC1 [24–26]. Thus, the ZNP-induced decrease in mTORC1 activity is likely responsible for the promotion of desmosomal cadherin endocytosis. Additionally, the successful rescue of ZNP-induced changes in vivo by 3BDO proved that mTORC1 is an efficient target for treating ZNP-induced epidermal damage. However, we do not yet know how mTORC1 participates in ZNP-induced desmosomal cadherin endocytosis.

TFEB is a well-known transcription factor downstream of mTORC1 that reportedly drives endocytosis by upregulating related genes [33]. However, it is unclear whether TFEB drives the endocytosis of desmosomal cadherins. Proteomic analysis of the phosphorylated proteins showed that the level of phosphorylated TFEB was drastically decreased after ZNP treatment. Immunoblotting and fluorescence analysis showed that ZNPs decreased p-TFEB/TFEB ratio and promoted the translocation of TFEB from cytoplasm to nucleus. These findings suggested that ZNPs can promote TFEB activation. Then, TFEB-siRNA was used to inhibit TFEB expression. The immunoblot results proved that the TFEB level in nuclear lysates was lower in TFEB-siRNA-treated cells than in nontargeting siRNA-treated cells. Moreover, in the presence of ZNPs, TFEB-siRNA decreased the levels of Dsg3 and Dsc3 in cytoplasmic lysates and increased the levels of Dsg3 and Dsc3 in cell membrane lysates. This finding suggested that TFEB participates in ZNP-induced desmosomal cadherin endocytosis. TFEB-siRNA also inhibited the ZNP-induced fragmentation of keratinocyte monolayers. Thus, in addition to mTORC1, TFEB is also a potential target for treating ZNP-induced epidermal damage. Unfortunately, the effect of TFEB inhibition was not tested in vivo for technical reasons. We failed to efficiently and noninvasively transfect siRNAs into keratinocytes in vivo.

As mentioned before, TFEB promotes endocytosis by upregulating related genes, and these genes have been predicted and summarized previously [33]. We checked which of these genes were also differentially expressed in response to ZNPs via proteomic analysis and found that BLOC1S3 was the only upregulated target and was upregulated 100-fold. BLOC1S3 is involved in the biogenesis of lysosome-related organelle complex 1 and plays a key role in intracellular vesicle trafficking [55, 56]. Vesicle trafficking is a necessary step in the transfer of the endocytosed cargos into the cytoplasm [57–59]. However, the role of BLOC1S3 in desmosomal cadherin endocytosis has not been determined. These immunoblot results were also in agreement with the proteomic results, indicating that ZNPs promote BLOC1S3 expression, which can be inhibited by TFEB-siRNA pretreatment. These findings indicate that ZNPs promote BLOC1S3 expression by activating TFEB. Then, BLOC1S3-siRNA was used to confirm the role of BLOC1S3 in ZNP-induced desmosomal cadherin endocytosis. BLOC1S3-siRNA partially inhibited the ZNP-induced endocytosis of Dsg3 and Dsc3. Nevertheless, BLOC1S3-siRNA was still found to be beneficial for preventing ZNP-induced fragmentation of the keratinocyte monolayer.

Notably, this study is based on a mouse model lacking a stratum corneum, unlike in previous studies, which used mice with an intact stratum corneum [6–8]. Although previous studies have shown that the stratum corneum prevents ZNPs from penetrating into the deep epidermis, the stratum corneum can be removed easily, and its function can be impaired in many ways, such as by excessive cleaning or frequent rubbing [9]. Skin inflammation reportedly promotes the penetration of ZNPs into deep epidermis and dermis despite the intact stratum corneum [10]. This finding suggested that stratum corneum does not reliably prevent the penetration of ZNPs into deep epidermis. In this study, the stratum corneum of mice was removed by silicon dioxide scrub cream instead of by excessive mechanical force or drugs that can induce skin disease because before ZNP exposure, a normal state of epidermis and keratinocytes was needed. The practical significance of this model is that excessive cleaning and minor abrasions are sufficient to remove the stratum corneum; under these conditions, ZNPs may increase the risk of epidermal fragmentation.

In fact, the biological events we have demonstrated in this study are only a small part of the biological effects of ZNPs. In addition to desmosomal cadherins, there are many other types of cadherins at the cell membrane. They are involved in intercellular junctions and act as signaling molecules in the regulation of the cell cycle, motility and differentiation [58–64]. The effects of ZNPs on these cadherins are not fully understood. Therefore, both an opportunity and a challenge exist in the biological application of ZNPs in the future. The more clearerly the biological effects of ZNPs are understood, the more likely they are to be used in appropriate fields and to avoid side effects.

## Conclusions

In summary, this work demonstrated that ZNPs can cause desmosome depolymerization and decreased epidermal mechanical strain resistance by promoting desmosomal cadherin endocytosis. Moreover, ZNP-induced desmosomal cadherin endocytosis was attributed to a decrease in mTORC1 activity, which promoted the dephosphorylation and nuclear translocation of TFEB. Activated TFEB significantly upregulated BLOC1S3, resulting in an increase in desmosomal cadherin endocytosis. These discoveries indicate that ZNPs can increase the risk of epidermal fragmentation and may provide potential targets for treating ZNP-induced epidermal damage.

## Data Availability

Data is provided within the manuscript.

## Abbreviations

3BDO: 3-Benzyl-5-(2-nitrophenoxymethyl)-gama-butyrolactone derivative
BLOC1S3: Biogenesis of lysosome-related organelle complex 1 subunit 3
DP: Desmoplakin
Dsg1: Desmoglein 1
Dsg3: Desmoglein 3
Dsc1: Desmocollin 1
Dsc3: Desmocollin 3
LC3B: Microtubule-associated protein 1 light chain 3 beta
mTORC1: Mammalian target of rapamycin complex 1
PG: Plakoglobin
PKP2: Plakophilin 2
RAPA: ReRapamycinac
S6K: Ribosomal protein S6 kinase
TFEB: Transcription factor EB
ZNP: Zinc oxide nanoparticle
